# High wettability of liquid caesium iodine with solid uranium dioxide

**DOI:** 10.1038/s41598-017-11774-0

**Published:** 2017-09-13

**Authors:** Ken Kurosaki, Masanori Suzuki, Masayoshi Uno, Hiroto Ishii, Masaya Kumagai, Keito Anada, Yukihiro Murakami, Yuji Ohishi, Hiroaki Muta, Toshihiro Tanaka, Shinsuke Yamanaka

**Affiliations:** 1 Graduate School of Engineering, Osaka University, 2-1 Yamadaoka, Suita, Osaka 565-0871 Japan; 2 JST, PRESTO, 4-1-8 Honcho, Kawaguchi, Saitama 332-0012 Japan; 3 Research Institute of Nuclear Engineering, University of Fukui, 1-2-4 Kanawacho, Tsuruga, Fukui 914-0055 Japan

## Abstract

In March 2011, the Fukushima Daiichi Nuclear Power Plant accident caused nuclear fuel to melt and the release of high-volatility fission products into the environment. Caesium and iodine caused environmental contamination and public exposure. Certain fission-product behaviours remain unclear. We found experimentally that liquid CsI disperses extremely favourably toward solid UO_2_, exhibiting a contact angle approaching zero. We further observed the presence of CsI several tens of micrometres below the surface of the solid UO_2_ sample, which would be caused by the infiltration of pores network by liquid CsI. Thus, volatile fission products released from molten nuclear fuels with complex internal composition and external structure migrate or evaporate to varying extents, depending on the nature of the solid–liquid interface and the fuel material surface, which becomes the pathway for the released fission products. Introducing the concept of the wettability of liquid chemical species of fission products in contact with solid fuels enabled developing accurate behavioural assessments of volatile fission products released by nuclear fuel.

## Introduction

In the severe accident in March 2011 at the Fukushima Daiichi Nuclear Power Plant (1F), the nuclear fuels containing fission products (FP) were exposed to a distinctive environment containing high-temperature water vapour^1–3^. Because of this exposure, portions of the core itself melted, and a large quantity of volatile FP, approximately one percent of the volume of materials released during the Chernobyl nuclear disaster, was released. This release caused contamination of the surrounding environment and public exposure to hazardous radioactivity^4^. Subsequently, to investigate the causes of the incident and stabilise the plant itself, various basic research efforts were launched to assess the fuel behaviour at the time of the incident^5^, to assess the behaviour of the radioactive materials released^6^, and to measure the physical properties of the molten fuel^7^. The highest priority issues among these research efforts include assessing the release behaviour of Cs and I, which are two highly volatile FP chemical species, the extent of Cs-related contamination of the surrounding environment, and public exposure to radioactive I. For example, both European and American research groups have reported on the statuses of atmospheric dispersion and soil surface accumulation of Xe and Cs released into the atmosphere during the 1F incident^8^.

However, even before the 1F incident, the release behaviour of various types of FP originating from molten fuels has been extensively researched through several out-of-pile large-scale comparative studies using irradiated fuel rods and fuel assemblies following the Three Mile Island No. 2 Reactor (TMI-2) incident in 1979^9^. In addition, the chemical equilibria of the chemical forms of Cs and I were calculated under various conditions, and the release behaviour of volatile FP has been discussed based on these results^10^. Furthermore, other studies have evaluated and analysed the migration and dispersal behaviour exhibited by FP contained in fuel^11^ and the behaviour of FP released from fuel^12^ through relatively small-scale testing methods, such as heating irradiated fuel samples directly.

We have learned from the results of these studies that to some extent for Cs and a much greater extent for I, the predominant chemical structure of these materials in nuclear fuel is CsI. These studies also have shown that CsI will switch from a solid to a liquid phase and eventually evaporate into a Cs- and I-bearing vapour phase that separates from the solid-fuel body in certain situations, including conditions when solid-fuel temperatures increase during a severe accident. However, we still have not gained a full understanding of the release behaviour exhibited by Cs and I during a severe accident. For example, as is summarised in greater detail in ref. 9, in out-of-pile tests, testing temperatures and time elapsed can vary. In these tests, release rates for Cs and I tended to increase when the materials were subject to hydrogen-rich atmospheric environments. However, a clear quantitative relationship has not been found between these test parameters and FP release rates.

Given this background, we believe that multiple factors, such as the wettability and solid–liquid interface properties of CsI relative to solid-fuel surfaces, must influence the degree of migration, evaporation, and even the behaviour of Cs and I released from solid fuels. Because molten fuel typically has a more complex internal structure and external shape than non-molten fuel, the surface ratio between solid fuels and liquid CsI becomes relatively large. In addition, when the surface contribution predominates, increase of lead concentration at the surface of copper–lead alloys^13^ and reduced melting temperature in various types of metallic nanoparticles^14^, an unusual combination of phenomena that is typically not observed in the bulk scale can be observed. In this study, we sought, through experimental testing, to evaluate both the wettability of liquid CsI toward uranium dioxide (UO_2_), a solid typically present in molten nuclear fuel, and the solid–liquid interface energy. This paper will also discuss, based on our results, both the form of liquid CsI present in molten fuel and the behaviour of Cs and I released from molten fuel.

## Results and Discussion

One method for assessing solid and liquid wettability is the sessile drop method. This method assesses wettability and solid–liquid interface energy based on the contact angle formed when a test liquid contacts the surface of a sufficiently smooth solid. This technique has been used for making precise assessments of the solid–liquid interface energy of compounds, such as solid-phase magnesium oxide and molten lead^15^. This study assessed the wettability of liquid CsI relative to solid UO_2_ using the sessile drop technique. Polycrystalline UO_2_ pellets, sufficiently polished to exhibit mirror-like smoothness, were selected as solid test materials. Then, CsI was melted onto the surface of these pellets. A series of tests were conducted using an electric furnace filled with high-purity argon gas, to minimise the possibility of oxidation of either UO_2_ or CsI during testing. The shape and wetting angle of the liquid phase were observed directly through holes in the walls of the electric furnace assembly. Please refer to the Methods section to review additional details of the tests using the sessile drop method.

In general, during sessile drop testing, the liquid sample has a constant contact angle relative to its placement on a solid substrate. If there are no chemical reactions between the two substances, Young’s relationship between the liquid surface energy *σ*_L_ and the interface energy between the solid and liquid substances *σ*_LS_ is obtained from the relation.

1 $${\sigma }_{{\rm{S}}}={\sigma }_{{\rm{LS}}}+{\sigma }_{{\rm{L}}}\,\cos \,\theta .$$

In Eq. (1), *σ*_S_ is the solid surface energy and *θ* represents the contact angle of the liquid placed onto the surface of the solid. In this study, the solid–liquid interface energy between solid UO_2_ and liquid CsI was assessed by direct observation and measurement of these contact angles.

However, we used the dihedral angle method, a technique based on an entirely different concept from sessile drop testing, to verify the validity of the results assessing the solid–liquid interface energy. In dihedral angular measurement testing, the solid–liquid interface energy *σ*_LS_ between solid UO_2_ and liquid CsI is calculated from the UO_2_ granular boundary angle formed by contact between the polycrystalline UO_2_ and liquid CsI, using the relation^16^.

2 $${\sigma }_{{\rm{LS}}}\,\cos \,\frac{{\theta }_{1}}{2}={\sigma }_{{\rm{S}}}\,\cos \,\frac{{\theta }_{2}}{2}.$$

It was confirmed by the EDX analysis that there was no diffusion of Cs or I into the UO_2_ grain at the sub-micron scale. Based on this experimental result, we used Eq. (2) on the assumption that the grain boundary energy of UO_2_ is the same below the CsI droplet and in a surface with no CsI. In Eq. (2), *σ*_S_ represents the UO_2_ surface energy (0.752 Jm^−2^ ^17^ at the liquid CsI melting temperature of 973 K) used in Eq. (1), and *θ*_1_ and *θ*_2_ represent the typical UO_2_ granular boundary angles coexisting with and without liquid CsI, respectively. Currently available research findings indicate the possibilities for precise assessment of the solid–liquid interface energy of compounds such as aluminium–tin eutectic crystals^16^. In this study, after crystal grains had grown sufficiently through heat pretreatment, the UO_2_ pellets were treated at 973 K for 48 h while liquid CsI permeated the surface. To inhibit the vaporization of liquid CsI, the treatment was performed in a sealed silica tube. Next, the pellets were quickly cooled to room temperature. After the CsI was completely removed, the microstructure of the UO_2_ pellets was observed using a scanning electron microscope (SEM). The same procedure was followed with UO_2_ pellets not permeated by CsI. Grain boundary angles were determined by measuring the angles created by the boundary formed by contact between two crystals at the points parallel and perpendicular to the observation plane. The resolution of the SEM images used for dihedral angle measurements is enough high, thus it is considered that the error in the measurement due to the limits in the resolution is within ±1°. Approximately 150 dihedral angles were observed for each sample material, and the dihedral angle appearing with the highest frequency was determined by statistically processing the observational data obtained.

The same tests were performed again using boron oxide (B_2_O_3_) instead of CsI to obtain reference data for the sessile drop tests. B_2_O_3_ was selected as a reference substance because it has a low melting point near the melting point of CsI, high chemical stability that permits easy handling and, as will be described subsequently, surface energy comparable to the surface energy of CsI. Additional characteristics of the UO_2_, CsI, and B_2_O_3_ samples are available in the Supplementary Information.

**Figure 1:**
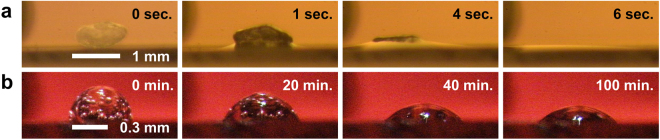
Melting factors for CsI and B_2_O_3_ added to solid UO_2_. (**a**) Liquid CsI immediately exhibits high wetting after melting onto the solid UO_2_ surface. (**b**) Liquid B_2_O_3_ maintains an almost hemispherical shape on the solid UO_2_ surface. Data are displayed chronologically from left to right.

The conditions of the CsI and B_2_O_3_ samples melted onto the surface of solid UO_2_ appear in Fig. 1(a) and (b), respectively. The melting conditions of the CsI and B_2_O_3_ samples as the electric furnace temperature increased were documented using video (Video 1 and Video 2 in the Supplementary materials). The liquid CsI exhibited melting behaviour close to its melting temperature of 900 K and subsequently displayed high wettability onto the solid UO_2_. As can be confirmed from the videos, the wetting kinetics of CsI is very fast, which is typical of the wetting of non-reactive liquids/solid couples for low viscosity liquids^18, 19^. The viscosity of molten CsI has been previously reported to be approximately 1.2 mPa·s at 973 K^20^. The contact angle between the solid UO_2_ and liquid CsI was virtually 0°. No reports or acknowledgements have been released to date regarding the high wettability of liquid CsI towards solid UO_2_. Thus, this behaviour constitutes a novel finding of our study. Note that here, the perfect wetting can be observed even if *θ* > 0° by the roughness effect expressed by Wenzel’s equation^18, 19^. However, we did not take into account the roughness effect in the contact angle measurements.

We recently have observed in various systems the same phenomenon, in which solids have a fluorite structure identical to UO_2_ and liquids are caesium halides like CsI. For instance, we have confirmed that liquid CsCl and CsBr show high wettability with single crystalline yttria-stabilized zirconia (YSZ)^21^. Video 3 (liquid CsCl on YSZ (100) plane) and Video 4 (liquid CsBr on YSZ (100) plane) in the Supplementary Materials provide additional illustrations of these findings.

B_2_O_3_ changed to a hemispherical shape after melting and then gradually wet the surface of the solid UO_2_. It is considered that bubbles in the B_2_O_3_ liquid come from pores that are contained in the starting materials. The contact angle varies with time and finally becomes a constant value. As an example, the variation of the contact angle with time for liquid B_2_O_3_ on the YSZ (100) plane is shown in the Supplementary Information. Differing from the behaviour of CsI, liquid B_2_O_3_ formed a contact angle of approximately 46° with the solid UO_2_. We confirmed by using SEM and transmission electron microscopy to observe in detail that the chemical reactivity between the UO_2_ and CsI, as well as between UO_2_ and B_2_O_3_, was vanishingly low. Low chemical reactivity between the solid-phase UO_2_ and liquid-phase CsI also was confirmed through thermodynamic equilibrium calculations. The Supplementary Information provides additional details of these determinations.

The contact angle between the liquid CsI and solid UO_2_ was nearly 0°, and no chemical reactions were observed between them. Therefore, we assumed that Young’s relationship would also hold in this situation, and we performed the calculation using Eq. (1) with *θ* = 0°. In this case, Eq. (1) is reduced to *σ*_LS_ = *σ*_S_ − *σ*_L_, known as the Antonoff law^22^, which has been widely applied to estimate internal energy between immiscible liquids. Values for the solid UO_2_ surface energy and liquid CsI surface energy values obtained from the literature^17, 23^ were used in this calculation. Using these results, an interfacial energy *σ*_LS_ of 0.69 Jm^−2^ between solid UO_2_ and liquid CsI was obtained. In the same manner, an interfacial energy of 0.74 Jm^−2^ between solid UO_2_ and liquid B_2_O_3_ was obtained using the contact angle (*θ* = 46°) as well as solid UO_2_ surface and liquid B_2_O_3_ surface energy values obtained from the literature^24^. Table 1 summarises the values used for these calculations.

**Table 1:** Figures for calculating solid–liquid interface energies.

	CsI (l)-UO_2_ (s)	B_2_O_3_ (l)-UO_2_ (s)	Reference
CsI or B_2_O_3_ melting temperature (K)	900	738	—
UO_2_ surface energy *σ*_S_ (Jm^−2^) at the melting temperature for each liquid	0.76	0.785	17
Liquid surface energy *σ*_L_ (Jm^−2^) at each liquid’s melting temperature	0.072	0.064	18, 19
Solid–liquid interface energy *σ*_SL_ (Jm^−2^) for each liquid	0.69	0.741	—
Solid–liquid adhesion *W* (Jm^−2^) for each liquid melting point	0.14	0.11	—

**Figure 2:**
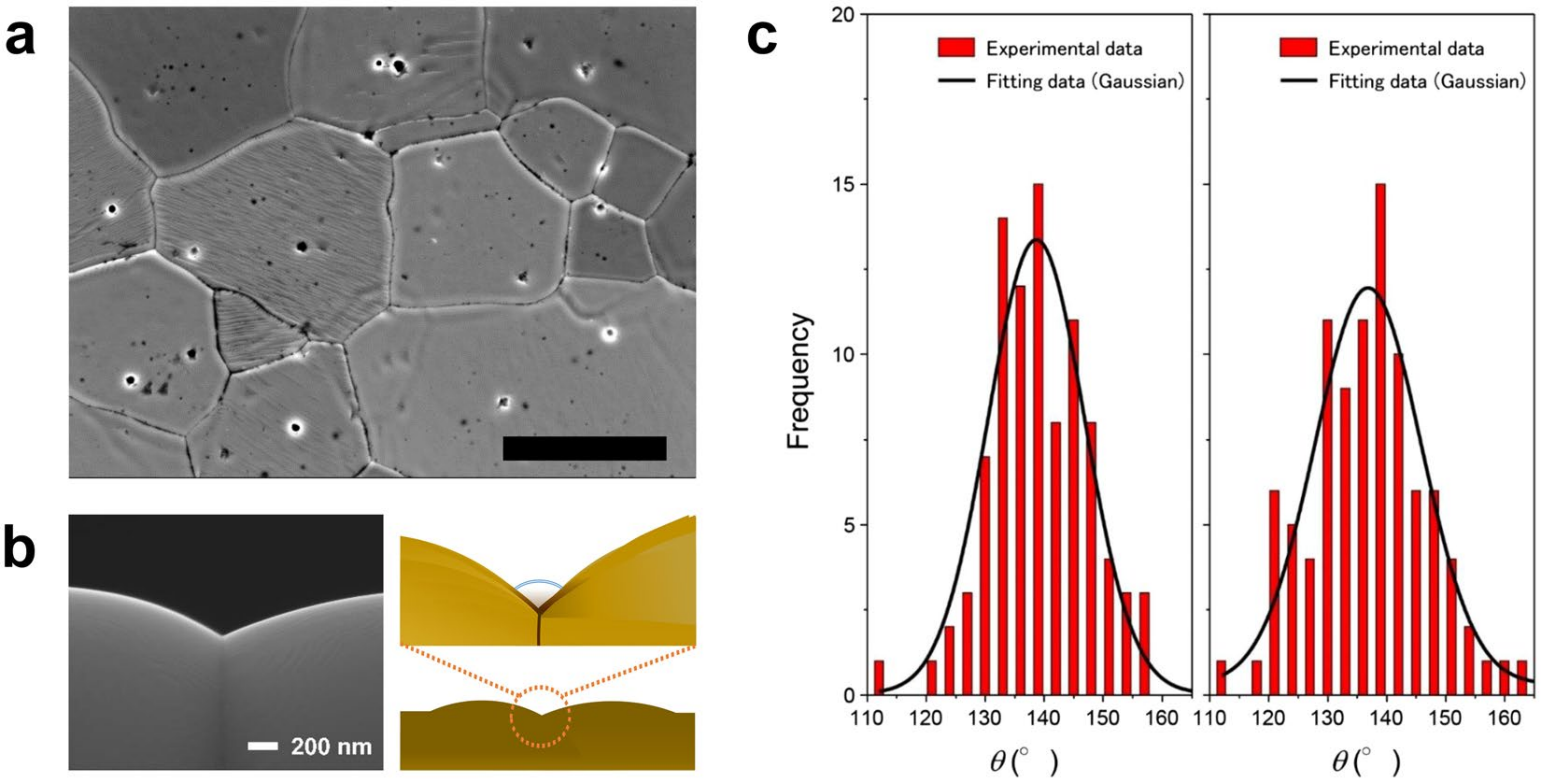
Dihedral angle method imagery and measurements. (**a**) SEM image of the surface of a UO_2_ pellet used in the dihedral angle method test. Scale bar is 10 μm. (**b**) An example of dihedral angle measurements depicted schematically and photographically. The SEM picture is from polycrystalline CeO_2_. (**c**) The grain boundary-angle distribution. Left: during liquid CsI penetration; and right: with unpenetrated UO_2_ pellets. The samples were at the maximum temperature of 973 K for 48 h. Here, the vertical red bars present experimental data and the solid black curves depict Gaussian fitting data. The mean values were calculated by fitting a Gaussian function. The selected dihedral angles exhibited the highest appearance frequencies. The standard deviation was approximately 11°.

Figure 2(a) displays SEM images of the grain boundaries on the surfaces of the liquid-CsI-wetted UO_2_ pellets used in the dihedral angle method tests. Using low magnification SEM images for the dihedral angle measurement may lead to significant errors^25^. Therefore, for accurate dihedral angle measurements, high resolution SEM images were used. As seen in these photographs, the crystal grains developed sufficiently, and grain boundaries can be clearly observed. In addition, Fig. 2(b) displays an example of dihedral angle measurements taken from a perpendicular observation perspective. The boundaries of 150 grains each of CsI-wetted and non-wetted UO_2_ were observed and measured following the same procedure, and the angular measurements were assessed to determine the frequencies of appearance, as shown in Fig. 2(c). It was confirmed that there are small humps at both sides of the grooves at the grain boundaries of polycrystalline UO_2_, which suggests that the grooves are growing via a diffusion controlled mechanism. In this case, the solubility of UO2 in liquid CsI is negligible. We confirmed that the grain boundary angles exhibited a distribution concentrated close to a specific mean value and calculated the mean value by fitting a Gaussian function to the data obtained. From these results, we determined that the dihedral angle *θ*_1_ for UO_2_ immersed with liquid CsI was 137°, and the dihedral angle *θ*_2_ for unwetted UO_2_ was 139°. When Eq. (2) was used to calculate the interfacial energy *σ*_LS_ between solid UO_2_ and liquid CsI based on these dihedral angle measurements, a value of 0.68 Jm^−2^ was obtained. This interfacial energy value between solid UO_2_ and liquid CsI obtained through the dihedral angle method was consistent with the value obtained by the sessile drop technique, a technique based on an entirely different concept. Therefore, one may state that this value has high validity.

We based our theory on the premise of the shift from solid–liquid wettability to adhesion, using the solid–liquid interface energy values obtained in this manner. In general, when a liquid adheres to a solid, the adhesion energy W of that liquid is determined from the relation^26^.

3 $$W={\sigma }_{{\rm{L}}}+{\sigma }_{{\rm{S}}}-{\sigma }_{{\rm{LS}}}.$$

We used this equation for calculating *W* between solid UO_2_ and liquid CsI and between solid UO_2_ and liquid B_2_O_3_, which were found to be 0.14 Jm^−2^ and 0.11 Jm^−2^, respectively. These results indicate that liquid CsI has greater adhesion towards UO_2_ than does B_2_O_3_, which demonstrates that more energy is required to detach the liquid from the solid because of the greater adhesion. The phenomenon of perfect or nearly perfect wetting of a non-reactive and immiscible liquid on a solid substrate is frequent at room temperature^27^, while it is definitely rare with high temperature liquids of metals, molten oxides, and salts^18, 19^. The very high wettability of UO_2_ by liquid CsI may be related with the very low *σ*_L_ value of liquid CsI (0.072 Jm^−2^), which is closer to the surface energy of room temperature liquids (normally tens of mJm^−2^)^27^ than to typical high temperature liquids (normally hundreds of mJm^−2^)^18, 19^. However, the surface energy of the liquid is not the only factor affecting wettability, as shown by the fact that while CsI and B_2_O_3_ have nearly the same surface energy, their contact angle differs significantly. This difference results from the fact that the adhesion energy of CsI on UO_2_ is higher than the adhesion energy of B_2_O_3_.

**Figure 3:**
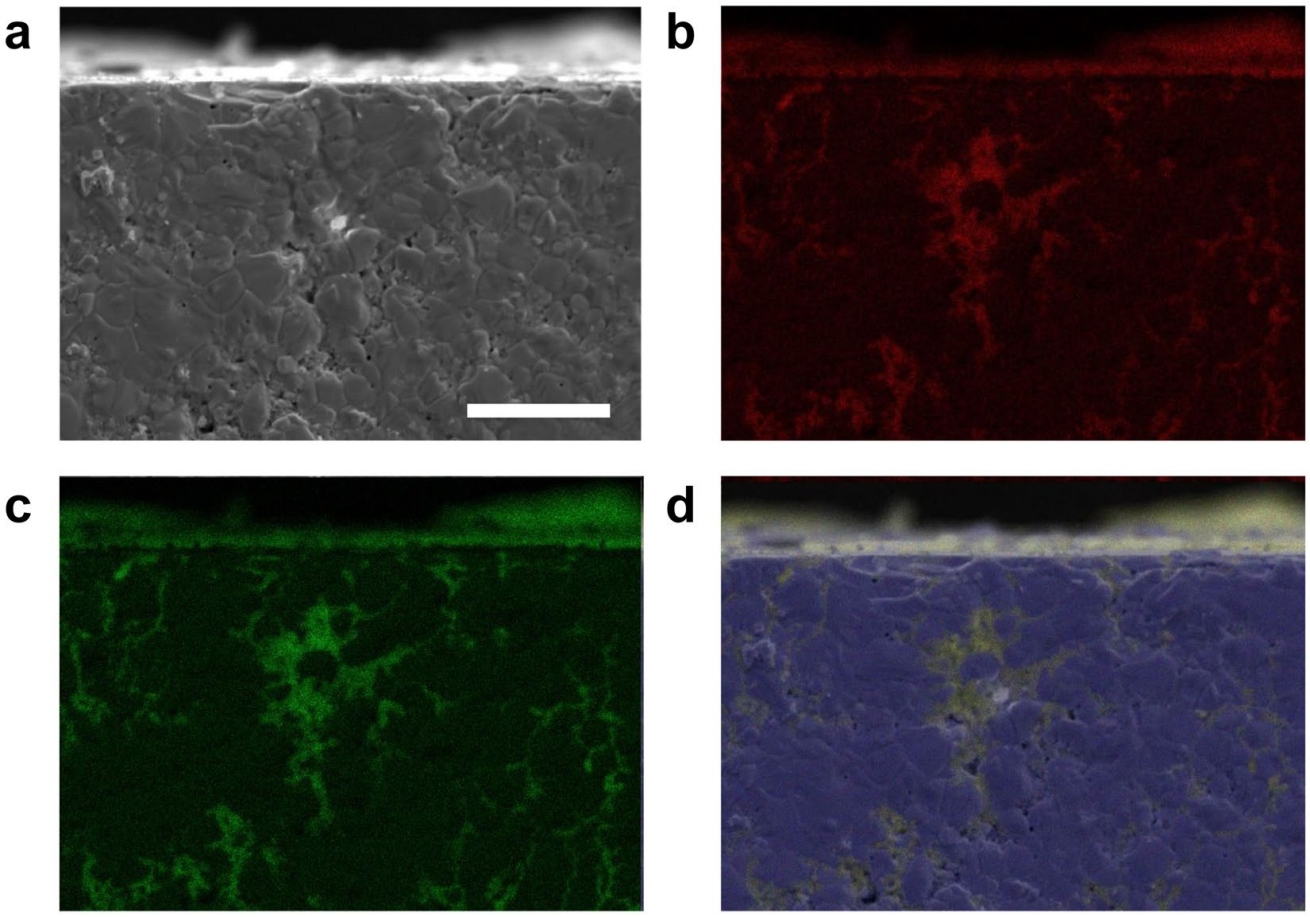
Cross-sectional observation results following sessile drop method testing between solid UO_2_ and liquid CsI. The sample was at the maximum temperature with liquid CsI for 1 minute in maximum. (**a**) SEM image and EDX mapping images of (**b**) I, (**c**) Cs and (**d**) U. Observations noted deep infiltration of liquid CsI into the solid UO_2_ on a macroscopic scale by way of the pores network present in the polycrystalline structure. Scale bar is 20 μm.

Next, Fig. 3 displays the results of our surface observations after sessile drop testing of liquid CsI in conjunction with solid UO_2_. First, as previously stated, traces of the chemical reaction between UO_2_ and CsI were not confirmed. Subsequently, we confirmed the presence of CsI several tens of micrometres below the surface of the solid UO_2_ sample used in testing. Because liquid CsI exhibits acceptable wettability toward solid UO_2_, CsI that has melted onto the surface of solid UO_2_ is believed to infiltrate inside the UO_2_ pellets via the pores network. Long range infiltration can occur if the two following conditions are fulfilled, (i) The contact angle *θ* must be much lower than 90°^28^ which is indeed the case for the couple CsI/UO_2_ for which a perfect or nearly perfect wetting was observed; and (ii) The pores fraction *a*_p_ must be higher than the critical value *a*_p_* for pores interconnection. For an isotropic solid with a dihedral angle *θ*_2_ ≈ 140°, the *a*_p_* is around 0.02^29^ which is lower than the experimental value *a*_p_ = 0.05 in the present case. In that case, the infiltration depth increases with time parabolically^28^. Such liquid-phase wetting phenomenon has been confirmed experimentally through demonstrations filling carbon nanotubes with molten lead by capillary action^30^, and penetration of polycrystalline alumina by glass at high temperatures^31^, but such demonstrations are extremely limited.

In the various experiments conducted to clarify the FP release behaviour of molten fuels^9–12^, numerous instances of incomplete release of Cs and I have been reported, even under conditions where the complete release of Cs and I would be normal. In reality, however, such irradiated fuel contains a diversity of phases^32^ and complex microstructure comprising numerous nanoscopic voids^33^. Because of this tendency, if liquid CsI appears in irradiated fuel, we can predict that it will be able to permeate the complex microstructure within the solid-fuel mass. In that case, the specific surface area of the liquid CsI becomes large, and thereby, the solid–liquid interface effects between solid UO_2_ and liquid CsI can be said to act to suppress FP release because of the high adhesion exhibited by liquid CsI with solid UO_2_. This phenomenon was not expected beforehand and will be beneficial in explaining the poorly understood behaviour of Cs and I released from fuel materials. In addition, this finding also constitutes a sufficient basis for re-examining the subject of numerous research projects: the behaviour of FP released from nuclear fuels during severe accidents.

## Methods

We used a special image of the reactor interior for our liquid-phase–wettability observations obtained using sessile drop testing. Using the device entailed placing the sample (UO_2_ pellet with CsI chunk or B_2_O_3_ chunk placed on top) onto a quartz plate fixed at the centre of an infrared heating apparatus. Then, a zoom-equipped complementary metal–oxide–semiconductor camera to the immediate right observed the shape of the liquid droplets on the sample surface. The camera had a maximum zoom of 300×. The sample temperature was measured using a radiation thermometer. Figure S1 in the Supplementary Information displays photographs of the vicinity of the sample plate component of the measurement apparatus.

As Figure S2 in the Supplementary Information illustrates, the diameter of a cylindrical UO_2_ pellet was approximately 9.5 mm, and its height was approximately 7.5 mm. These pellets were cut into disc-like pieces measuring 4 mm × 4 mm × 2 mm for testing. Randomly shaped chunks of either CsI or B_2_O_3_, with masses of approximately 1 mg or 0.3 mg, respectively, were placed on top of cut discs of UO_2_. The rate of temperature increase was set to rise from room temperature at a rate of 20 K/min to just under the melting temperature and then at 1 K/min near the melting temperature. The temperature was fixed after the samples had melted fully. The samples were photographed continuously at a rate of 10 photographs/s, and the most representative images were selected for use in obtaining angular measurements. Testing using both CsI and B_2_O_3_ was performed in an argon atmosphere. During angular measurements, photographs focused on the UO_2_ pellet surfaces. Alternately, composite photographs of the molten CsI or B_2_O_3_ were used. The nature of the surfaces of the liquid-phase CsI or B_2_O_3_ present on the UO_2_ pellets could be observed clearly through this approach. We took precise angular measurements. Representative angular measurements are displayed in Figure S3 in the Supplementary Information.

## Electronic supplementary material

Supplementary Information

Video 1

Video 2

Video 3

Video 4
