# Supplementary material for: High wettability of liquid caesium iodine with solid uranium dioxide
Source: Sci Rep. 2017 Sep 13;7:11449. doi: 10.1038/s41598-017-11774-0 (PMC5597628; doi:10.1038/s41598-017-11774-0)
Supplement: Supplementary file 1 — Supplementary Information [file 41598_2017_11774_MOESM1_ESM.pdf]

Supplementary Information

High wettability of liquid caesium iodine with solid uranium dioxide

Ken Kurosaki[†,‡], Masanori Suzuki[†], Masayoshi Uno[§], Hiroto Ishii[†], Masaya Kumagai[†], Keito Anada[†], Yukihiro Murakami[§], Yuji Ohishi[†], Hiroaki Muta[†], Toshihiro Tanaka[†], Shinsuke Yamanaka[†,§]

[†]Graduate School of Engineering, Osaka University, 2-1 Yamadaoka, Suita, Osaka 565-0871, Japan.

[‡]JST, PRESTO, 4-1-8 Honcho, Kawaguchi, Saitama 332-0012, Japan.

[§]Research Institute of Nuclear Engineering, University of Fukui, 1-2-4 Kanawacho, Tsuruga, Fukui 914-0055, Japan.

Corresponding author: Ken Kurosaki, kurosaki@see.eng.osaka-u.ac.jp

List of Videos

Video 1. Melting behaviour of CsI on polycrystalline UO_2.

Video 2. Melting behaviour of B_2O_3 on polycrystalline UO_2.

Video 3. Melting behaviour of CsCl on YSZ single crystal (100) plane.

Video 4. Melting behaviour of CsBr on YSZ single crystal (100) plane.

Preprocessing of UO_2 pellets

A predetermined preprocessing methodology was applied to UO_2 pellets used for both sessile drop method testing and dihedral angle method testing. Testing employed UO_2 pellets, containing depleted uranium with a density of approximately 95% T.D., which were produced by a manufacturer of nuclear fuel. Several disc-shaped samples, measuring 4 mm × 4 mm × 2 mm, were made from UO_2 pellets with diameters and heights of approximately 9.5 mm and 7.5 mm, respectively, for use in sessile drop testing. In addition, 2 mm cubes were made for dihedral angle

testing. After we polished the surfaces of the cut samples using a No. 2000 rough polishing sheet, a diamond polishing sheet (Maltose Co., Ltd.) was used to polish mirror surfaces to 3.0 μm, 1.0 μm, 0.5 μm, and 0.1 μm. Precise measurements of the granular boundaries were needed for dihedral angle testing. As such, the polished samples were subjected to heat treatment at 1023 K for 1 h for tension reduction and then were treated at 1723 K for 20 h to allow granular boundaries to develop. These heat treatments were conducted in a vacuum. A single UO_2 pellet was powdered after heat treatment and underwent powdered X-ray diffraction (XRD) analysis. Figure S4 displays the XRD pattern data obtained. Samples were single-phase UO_2 cubic crystals with a fluorite structure, and no prominent peaks caused by impurities were observed. The lattice constant calculated from this XRD pattern was 0.5487 nm, which was consistent with the previously reported lattice constant for $UO_{2.00}$ of 0.5480 nm.

Properties of CsI and B_2O_3 testing samples

Powder XRD pattern data for CsI and B_2O_3 used in sessile drop testing and dihedral angle method testing are displayed in Figures S5 and S6, respectively. For both CsI and B_2O_3, peak position and strength values calculated from the crystal structure were consistent. The samples were confirmed to be single phase and without impurities. The lattice constant for CsI was 0.4567 nm, which was consistent with the previously reported value of 0.4568 nm. We were unable to calculate an accurate lattice constant for B_2O_3 because a portion of the sample was amorphous.

Assessment of the chemical reactivity between UO_2 and CsI during sessile drop testing

Reactivity between UO_2 and CsI was evaluated by calculating the chemical equilibrium for the U-Cs-I-O series. Thermodynamic data for each phase exhibited by the chemical species considered for these calculations were obtained from the thermodynamic database of the U-Zr-Ce-Fe-B-C-I-O-H system originally developed by the authors' group. Table S1 displays the potential solution phase considered and stoichiometric compound phase data for the thermodynamic calculations. In addition, results of our chemical equilibrium calculations are displayed in Fig. S7. In this figure, the horizontal axis represents temperature, and the vertical axis represents the compound phase-weight at each temperature level. We can see from this figure that oxide compounds

comprising Cs and U, such as Cs_2UO_4 should not be generated. Even though our calculations accounted for various compound phases, liquid-phase CsI did not react with UO_2, and liquid CsI weight volatility decreased at its boiling point. Instead, this weight was increasingly replaced with gas-phase CsI. Based on these calculations, we confirmed that solid UO_2 and liquid CsI exhibited extremely low reactivity.

Table and Figures

Table S1. Solution and compound phases for U-Cs-I-O chemical equilibrium calculations

Solution phase	Gas real<br>(CsI, CsO, I_2, O_2, UI_4, UI_3, U, I, I_2, and Cs are included)
	Liquid iodine<br>(CsI, Cs, U, I_2, UI_4, and UI_3 are included)
	Liquid oxide<br>(Cs_2O, Cs, CsO_2, U, UO_2, and O are included)
	High temperature UO_{2+x} solid
Stoichiometric compound phase	CsI (s)
	Cs_2O (s)
	UO_2 (s1, low temperature)
	U_4O_9 (s)
	U_3O_8 (s)
	$Cs_2U_4O_{12}$ (s)
	Cs_2UO_4 (s)
	$Cs_2U_2O_7$ (s)
	$Cs_4U_5O_{17}$ (s)
	$Cs_2U_4O_{13}$ (s)
	$Cs_2U_6O_{18}$ (s)
	$Cs_4U_2O_7$ (s)
	$Cs_2U_5O_{16}$ (s)
	$Cs_2U_7O_{22}$ (s)
	$Cs_2U_9O_{27}$ (s)
	$Cs_2U_{15}O_{46}$ (s)

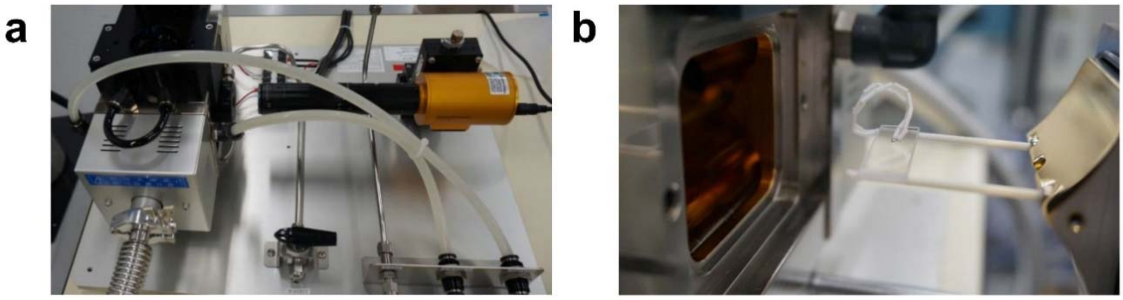

Figure S1. Equipment configurations for sessile drop testing. (a) The sessile drop measurement equipment. (b) The sample assembly surface.

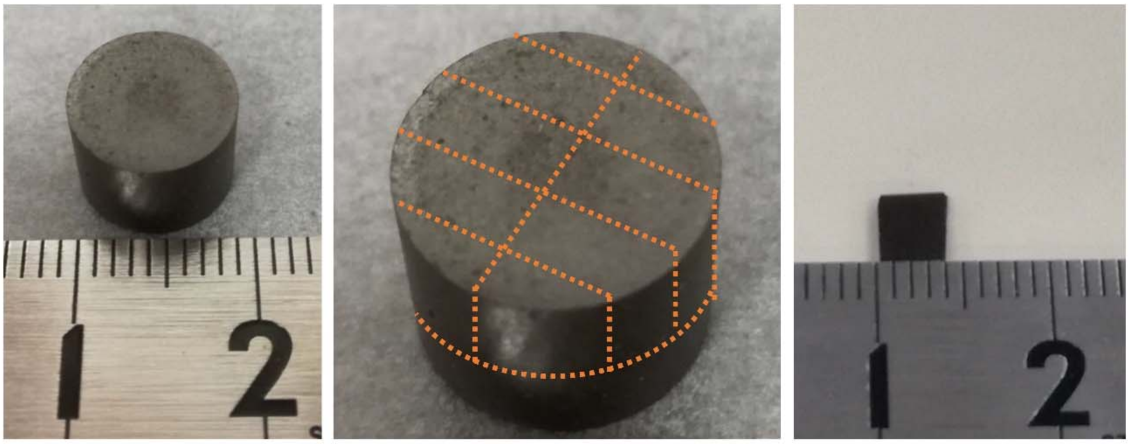

Figure S2. External photographs of UO_2 pellets and UO_2 panels used during sessile drop testing.

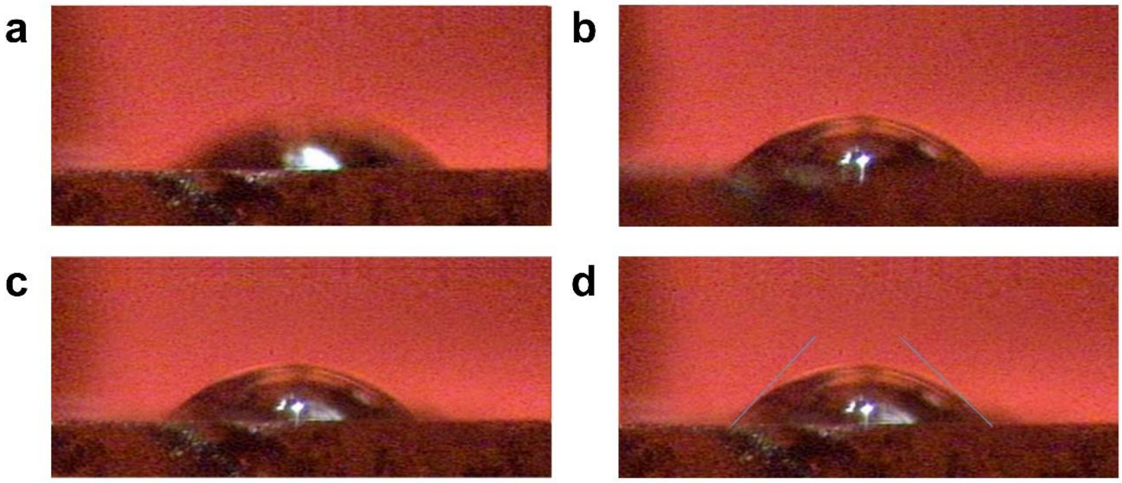

Figure S3. Example of liquid B_2O_3 contact angle measurement on the surface of a UO_2 pellet. Photographs of (a) the surface of the UO_2 pellet, (b) molten B_2O_3, (c) fused compound and (d) an example of the angular measurement.

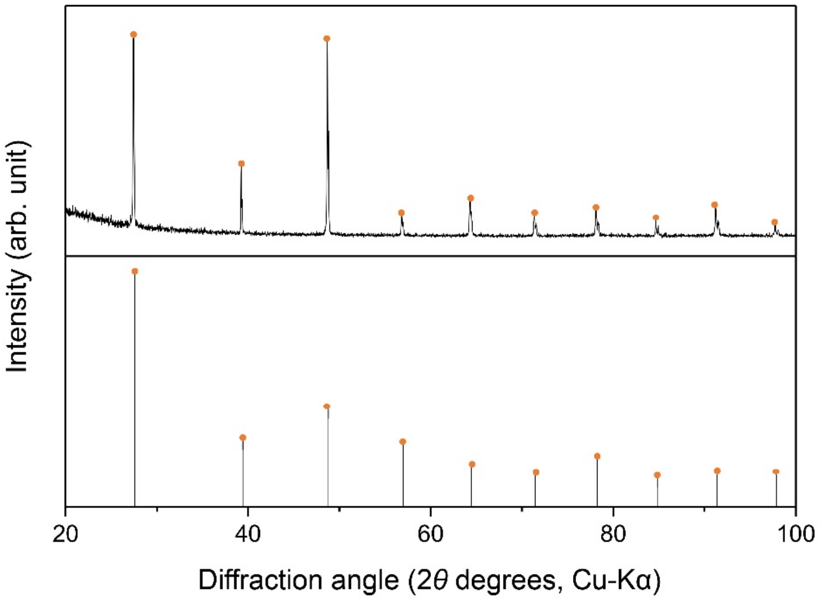

Figure S4. XRD pattern of UO_2 pellets used during dihedral angle method testing.

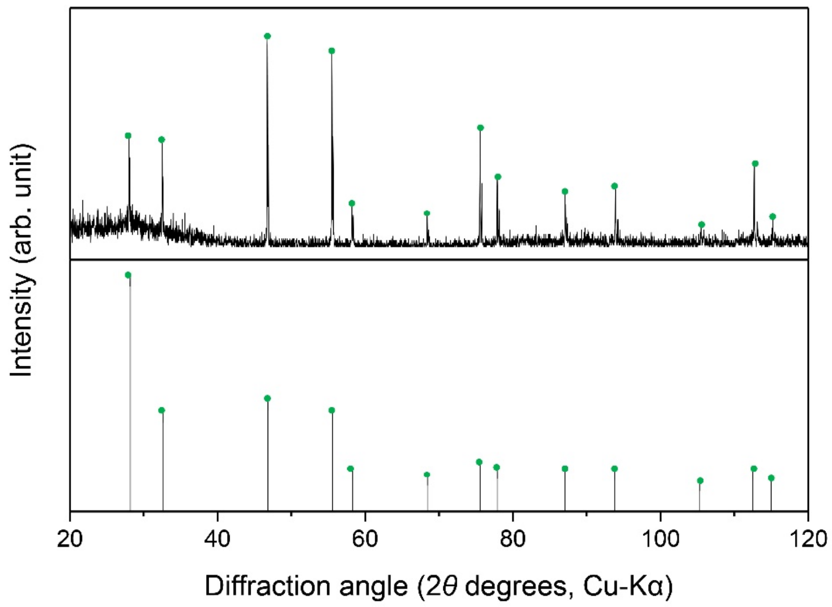

Figure S5. XRD pattern of powdered CsI used during dihedral angle method testing.

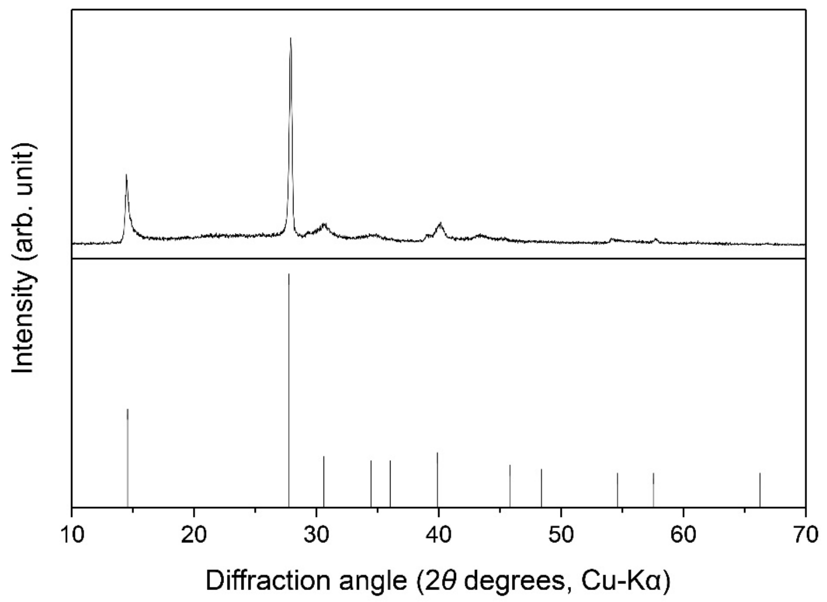

Figure S6. XRD pattern of powdered B_2O_3 used during sessile drop testing or dihedral angle method testing.

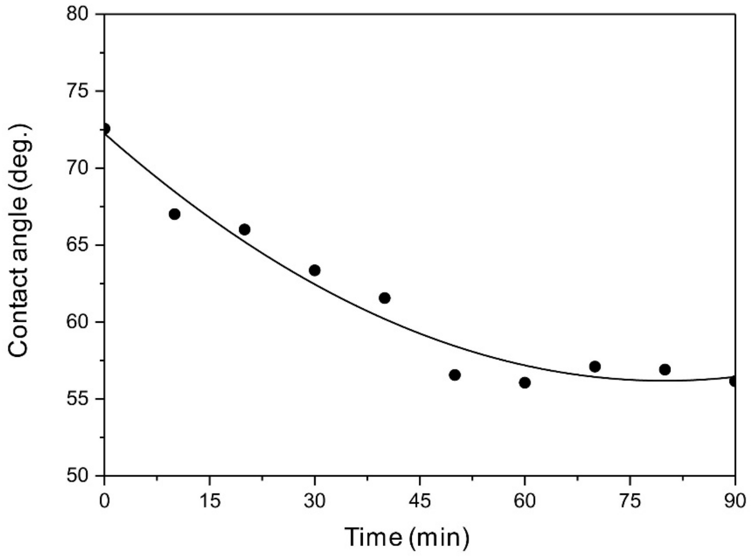

Figure S7. Variation of the contact angle with time for liquid B_2O_3 on YSZ (100) plane.

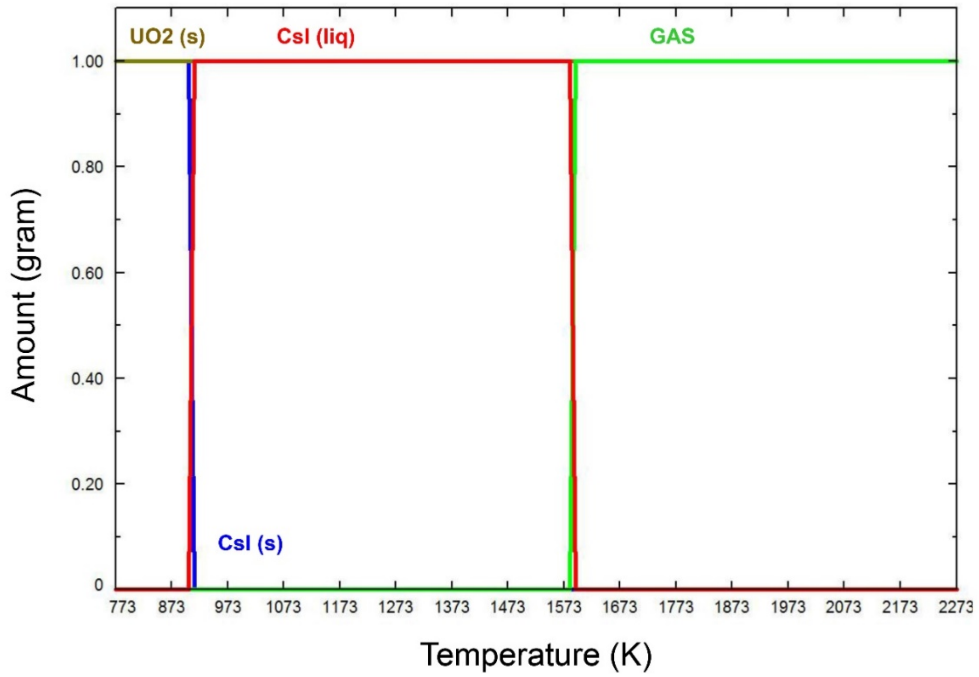

Figure S8. Results of chemical equilibrium calculations examining reactivity between solid UO_2 and liquid CsI. CsI occupies the GAS phase as the most dominant chemical species.
